# Comparison of Three Potassium Binders in Patients With Acute Hyperkalemia

**DOI:** 10.7759/cureus.86922

**Published:** 2025-06-28

**Authors:** Amy C Bower, Tracey L Mersfelder, Gregory S Wellman

**Affiliations:** 1 Inpatient Pharmacy, Ascension Borgess Hospital, Kalamazoo, USA; 2 Pharmacy, Ferris State University, Grand Rapids, USA; 3 Pharmaceutical Science, Ferris State University, Big Rapids, USA

**Keywords:** acute hyperkalemia, patiromer, potassium binders, sodium polystyrene sulfonate, sodium zirconium cyclosilicate

## Abstract

Background: Hyperkalemia is a common electrolyte abnormality that can occur in hospitalized patients, particularly those with chronic kidney disease. If not appropriately treated, hyperkalemia can result in life-threatening arrhythmias. While sodium polystyrene sulfonate (SPS), patiromer, and sodium zirconium cyclosilicate (SZC) are widely used potassium binders, no study has compared all three binders in lowering potassium in acute hyperkalemia in the acute care setting.

Objective: The primary outcome was the post-treatment change in serum potassium concentration, compared by binder, within 24 hours.

Methods: This retrospective cohort quality improvement project included 75 adult inpatients who received SPS, SZC, or patiromer at a community teaching hospital between 2021 and 2023.

Results: All three potassium binders showed a statistically significant difference in lowering potassium concentrations within 24 hours (p<0.001). Controlling for baseline potassium, standard of care, and BMI, SPS and SZC were superior to patiromer (p=0.001 and p=0.022, respectively). There was no statistically significant difference between SPS and SZC (p=0.206).

Conclusion: SPS and SZC were superior to patiromer in lowering serum potassium levels in patients with acute hyperkalemia, with no significant difference between SPS and SZC. Considering the established safety concerns associated with SPS, SZC may be the preferred binder for managing acute hyperkalemia in the inpatient setting.

## Introduction

Hyperkalemia is a common electrolyte disturbance in the acute care setting. High serum potassium (defined as ≥5 mmol/L to ≥6 mmol/L) occurs in approximately 2.9%-6.3% of hospitalized patients and up to 24.3% of those patients with acute kidney injury [1-3]. Traditional agents, such as sodium polystyrene sulfonate (SPS), have overall poor tolerability and an unpredictable onset of action [4]. Due to the potential for significant adverse events associated with SPS and calcium polystyrene sulfonate (CPS), including colonic necrosis, there is a need for alternative treatment options in patients with hyperkalemia [5,6]. Over the past 10 years, additional potassium binders, including sodium zirconium cyclosilicate (SZC) and patiromer, have been approved. Retrospective studies have been conducted to determine whether one potassium binder is more efficacious and safer than another [7-14]. To date, none of these studies have evaluated all three potassium binders available in the United States in a single study. This quality improvement study was designed to determine whether one potassium binder is superior to other binders in lowering serum potassium for the treatment of acute hyperkalemia in the inpatient setting. The primary outcome was the net change in serum potassium from baseline within 24 hours, with the a priori hypothesis that SPS and SZC would demonstrate a greater reduction in serum potassium compared to patiromer. The study’s secondary outcomes included the number of patients requiring rescue hemodialysis to correct serum potassium, average length of hospital stay after the incidence of hyperkalemia resolved, and the adverse events associated with each potassium binder, including edema and/or gastrointestinal intolerance, to explore safety outcomes.

## Materials and methods

This retrospective cohort study took place at a 435-bed community teaching hospital. Patients were included if they were 18 years or older, had a baseline serum potassium concentration ≥ 5.5 mmol/L before a potassium binder was administered, and received one dose of SPS, patiromer, or SZC with or without hyperkalemia standard of care. Standard of care could include regular insulin 5 units or 0.1 unit/kg (maximum of 10 units) intravenous (IV) push, dextrose 50% 25 mL or 50 mL IV push, calcium gluconate 1 g IV piggyback, albuterol 10 mg nebulized, or furosemide 40 mg or 80 mg IV push. A total of 75 patients were included in this study, with 25 patients in each arm.

Patients were excluded if they had a gastrointestinal motility disorder (i.e., ileus or bowel obstruction), underwent recent bowel surgery (≤3 months), received intermittent hemodialysis/continuous renal replacement therapy, or had end-stage renal disease at baseline; were pregnant; were prisoners; had diabetic ketoacidosis upon admission; received multiple different potassium binders within the treatment period; used a potassium binder 48 hours before the treatment period; had hemolyzed blood samples; or were on chronic/maintenance potassium binder therapy. Baseline characteristics for each patient were recorded on a data collection sheet including age, sex, body mass index (BMI), past medical history, and baseline serum potassium concentration before a binder is initiated. Serum potassium concentrations over 24 hours, length of stay, and electrocardiograph changes (ECG) were also recorded on the data collection sheet. ECG changes were defined as the presence of peaked T waves, a loss of the P wave, or a prolonged QRS complex in patients. Maintenance medications that had the potential to increase potassium at baseline, such as angiotensin-converting enzyme inhibitors (ACEIs), angiotensin receptor blockers (ARBs), angiotensin receptor/neprilysin inhibitors (ARNIs), mineralocorticoid receptor antagonists (MRAs), sulfamethoxazole/trimethoprim, and maintenance potassium supplements (IV or oral), were collected as well. Data collection began following approval by the Institutional Review Board (IRB) as a quality improvement project.

Data were pulled by the informatics pharmacist for the following periods: patients who received SPS from September 2021 to September 2023, patients who received patiromer from September 2021 to September 2022, and patients who received SZC from September 2022 to September 2023. These dates were selected based on the hospital formulary.

With a balanced design of 25 subjects per group and an estimated mean variance of 0.23, there was a power of 0.91 to detect a difference of 0.5 mmol/L across the three treatment groups. The primary outcome was a post-treatment serum potassium concentration comparison by the treatment group. Analysis of covariance (ANCOVA) was performed on the primary outcome, comparing the differences between post-treatment potassium for each treatment group, controlling for baseline serum potassium concentration, standard of care, and BMI. The additional outcome was paired comparisons of baseline serum potassium concentration versus post-treatment serum potassium concentration within each treatment group using the Wilcoxon signed-rank test. All statistical analyses were performed using Stata 18.0 (StataCorp LLC, College Station, TX).

## Results

The baseline characteristics were similar among the potassium binder groups, except that they differed significantly by sex. The SPS and patiromer treatment groups contained more male patients than the SZC treatment group. The mean age among the potassium binder treatment groups was 70 years old, the mean BMI was 31 kg/m^2^, and the mean baseline serum potassium concentration was 5.8 mmol/L (Table 1).

**Table 1:** Baseline Characteristics Abbreviations: BMI = body mass index; SD = standard deviation; SPS = sodium polystyrene sulfonate; SZC = sodium zirconium cyclosilicate

Potassium Binder Group	Mean Age (Years)	Sex (M: Male; F: Female)	Mean BMI (kg/m^2^)	Mean Baseline Potassium (mmol/L)
SPS	66	M: 22; F: 3	31 (SD 5.93)	5.8 (SD 0.52)
Patiromer	73	M: 18; F: 7	30 (SD 7.76)	6.0 (SD 0.49)
SZC	71	M: 12; F:13	32 (SD 12.69)	5.8 (SD 0.43)
p-value	0.104	0.009	0.710	0.167

Baseline maintenance medications and standards of care

Sixty percent (n=15) of patients in both the SPS and SZC groups were on therapy that could potentially increase serum potassium concentrations, compared to 88% (n=22) in the patiromer group. This difference was not statistically significant between the groups (p=0.063). The most common medications observed were ACEIs, ARBs, and ARNIs at 51%, potassium supplements at 32%, and MRAs at 27%. Standard of care for treatment of hyperkalemia could include regular insulin 5 units or 0.1 units/kg (maximum 10 units IV push, dextrose 50% 25 mL or 50 mL IV push, calcium gluconate 1 g IV piggyback, nebulized albuterol 10 mg, or furosemide 40 mg or 80 mg IV push. In the SPS group, 60% (n=15) received at least one medication classified as standard of care. In comparison, 68% (n=17) in the patiromer group and 64% (n=16) in the SZC group received at least one standard-of-care medication. These differences were not statistically significant.

ECG changes

There were 24% (n=6) of patients in the SPS group who experienced ECG changes, 16% (n=4) in the patiromer group, and 28% (n=7) in the SZC group. This did not significantly differ among the potassium binder treatment groups (p=0.587).

Primary outcome

The serum potassium concentrations for each of the three treatment groups are shown in Table 2. The net change in serum potassium concentrations (pre- to post-treatment) was greatest for SPS, followed by SZC, and least for patiromer. Paired comparisons within each of the treatment groups demonstrated significant reductions in serum potassium concentrations from baseline to post-treatment (p<0.001). 

**Table 2:** Summary of Potassium Concentrations (mmol/L) Before and After Treatment ^a ^Mean and (standard deviation); ^b^p-value from the Wilcoxon signed rank test comparing pre- to post-potassium levels Abbreviations: SD = standard deviation

Variables	Baseline Potassium		After Treatment Potassium		Before to After	
Binder	Mean (SD)	95% CI	Mean (SD)	95% CI	Net Change^a^	p-value^b^
Sodium polystyrene sulfonate (0) (n=25)	5.84 (0.52)	(5.63, 6.06)	4.68 (0.42)	(4.51, 4.85)	1.16 (0.74)	p < 0.001
Patiromer (1) (n = 25)	6.06 (0.48)	(5.86, 6.26)	5.30 (0.72)	(5.00, 5.59)	0.76 (0.63)	p < 0.001
Sodium zirconium cyclosilicate (2) (n = 25)	5.83 (0.43)	(5.66, 6.01)	4.91 (0.63)	(4.65, 5.17)	0.92 (0.85)	p < 0.001

The ANCOVA model revealed a statistically significant effect of the treatment group on post-treatment serum potassium concentrations (F (5, 69)=3.84, p=0.004), controlling for baseline serum potassium concentrations, standard of care, and BMI. The model explained 21.8% of the variability in post-treatment potassium (adjusted R²=16.1%). Compared to the reference group (patiromer), patients in the SPS group exhibited a significantly lower post-treatment serum potassium concentration (β=-0.621, p=0.001), controlling for the additional variables. Compared to the reference group (patiromer), patients in the SZC group also exhibited a significantly lower post-treatment serum potassium concentration (β=-0.405, p=0.022), controlling for the additional variables. In post-hoc analysis, SPS was not statistically significantly different from SZC in lowering post-treatment serum potassium concentrations (β=- 0.215, p=0.206). Residual diagnostic checks confirmed that the assumptions of normality (Shapiro-Wilk on residuals p=0.474) and linearity (p=0.560) were adequately met. Variance inflation factors were low (VIF: 1.02-1.40), indicating minimal multicollinearity among predictors.

Additional outcomes

Hemodialysis was not required in either the SZC or the SPS treatment groups to correct hyperkalemia. Twelve percent (n=3) of patients in the patiromer group received hemodialysis to correct their serum potassium concentration. This was a statistically significant difference (p=0.044).

No statistically significant difference was observed among the potassium binder groups regarding the length of stay. Most patients had an inpatient stay lasting longer than 72 hours.

Adverse events

Only 8% (n=2) of patients in the patiromer group experienced adverse events, which included edema and/or gastrointestinal intolerance (p=0.128). Neither the patients in the SZC nor the SPS treatment groups experienced adverse events.

## Discussion

Our study is the first, to our knowledge, to retrospectively evaluate the use of three different potassium binders in the same institution. Additionally, it is the first study to include patiromer as one of the treatment arms. These study designs strengthen our results and the ability to recommend one regimen over another in hospitalized patients with hyperkalemia.

Previous studies have evaluated the efficacy of the potassium-lowering capabilities of SPS and SZC in hospitalized patients [7,8,10-12,14]. One study used CPS as the comparator medication, and one study evaluated patients in the emergency department [9,13].

Four studies assessed the difference between SPS and SZC. Patients could receive other potassium-lowering therapies if the provider determined them to be necessary. All four studies determined that there was no difference in serum potassium at the next laboratory draw [13], within 24 hours [7,10], or at 48 hours [12].

One study evaluating SPS and SZC in the hospital setting also showed equal efficacy between the two treatments at all endpoints except at the eighth hour [11]. At hour eight, SZC lowered serum potassium to a larger degree from baseline compared to SPS (p=0.03) and had a lower serum potassium concentration (p=0.01).

Two studies that assessed SPS and SZC in the hospital setting had different results compared to the previously mentioned study [8,14]. Both concluded that SPS was more efficacious in decreasing serum potassium concentrations than SZC. Sullivan and colleagues evaluated the serum potassium concentrations four to 12 hours after treatment with 10 g of SZC or 15-30 g of SPS [8]. More patients in the SPS group achieved normokalemia (74.9%) compared to the SZC group (68.8%) (p<0.001). Lewis et al. evaluated the serum potassium concentrations 12-30 hours after treatment with a single dose of SZC 10 g or SPS 15-30 g [14]. More patients in the SPS group (69.2%) were normokalaemic compared to the SZC group (58.1%) within the timeframe of the study (p<0.01). Additionally, the average decrease in serum potassium was greater in the SPS group (0.99 mmol/L) compared to the SZC group (0.71 mmol/L) (p<0.01).

These studies were similar to ours concerning the patient population chosen to study the efficacy of potassium binders. The methods were also similar, with patients being able to receive additional treatments for hyperkalemia based on provider preference. The results of our study agreed with the conclusions of these studies in that SZC was not superior to SPS. All of these factors add strength to our study. Additional strengths of our study were that it included a third treatment arm, patiromer, and the fact that it was conducted in one institution that would have similar regional practices.

Four studies commented on safety and adverse events. Three studies evaluated the risk of hypomagnesemia [8,10,12]. Two among them concluded that SPS resulted in more hypomagnesemia than SZC [8,10]. One study had a 5.3% higher rate of hypomagnesemia in the SPS group, while the other study had only 0.9%. The third study concluded that SZC resulted in more hypomagnesemia by 4.6% compared to SPS [12]. Four studies commented on gastrointestinal (GI) adverse events in the studies [8,10,12,14]. In one study, there was one case of intestinal perforation in the SPS group and one case of bowel ischemia in the SZC group [8]. The authors concluded that the patients had other potential causes for these events, such as peritoneal metastasis and septic shock. A second study reported GI ulceration in two patients (1.7%) and intestinal colitis in three patients (2.5%) in the SPS group [10]. The authors concluded that these events could have been due to the medication. A third study had one patient who had colonic necrosis in the SZC group [12]. The authors did not believe that this event was due to the medication. Finally, one study reported no GI events in either group [14].

In our study, only 8% (n=2) of patients in the patiromer group had a documented adverse drug reaction. Neither the SZC nor the SPS groups experienced adverse reactions, including no cases of colonic necrosis. It can be challenging to find documentation of such reactions due to a combination of factors, including incomplete or inconsistent documentation and potential underreporting. Additionally, our study was not powered to detect a difference in the adverse effects of the treatment medications.

One of the limitations of this study was that other treatments for hyperkalemia were allowed, and, therefore, the independent effect of each therapy could not be determined due to this confounding variable. Additionally, there was no set time to evaluate the follow-up potassium concentration after administration of the study medications that demonstrate different pharmacodynamics. However, the design of our study reflects the real-world treatment of hyperkalemia regarding both of these points and was consistent with other literature [2,7-10,12-14]. Another limitation was that we did not take into account the cause of the hyperkalemia, which may affect the rate of achieving normokalemia. Subsequently, with the retrospective design of the project, we were unable to control for the prescribing preferences of the providers in each particular patient. Finally, the study was not powered to detect a difference in confounding variables.

A randomized controlled trial is ongoing to definitively answer the question of whether one potassium binder is superior to others and if any confounding variables may impact efficacy [15].

## Conclusions

The treatment of hyperkalemia has evolved in the past 10 years due to the approval of additional oral potassium binders. Many studies have attempted to determine if one potassium binder is superior to another. This answer would be important for decisions regarding hospital formularies. Our results showed that SPS and SZC, in addition to the standard of care, was were superior to patiromer in treating acute hyperkalemia. There was no significant difference between SPS and SZC in lowering serum potassium concentrations within 24 hours. Even though our study did not find any differences in the side effects of the three potassium binders, the adverse effects of SPS are well documentedwell-documented. These side effects are cause to question SPS as a standard in the treatment of hyperkalemia. When comparing the SZC and patiromer in addition to standard of care, SZC was found to be superior to patiromer and thus may be considered as the preferred formulary agent for the management of acute hyperkalemia in the acute care setting.
